# Autoantibodies versus Skin Fibrosis Extent in Systemic Sclerosis: A Case-Control Study of Inverted Phenotypes

**DOI:** 10.3390/diagnostics12051067

**Published:** 2022-04-24

**Authors:** Ashley Tieu, Benjamin Chaigne, Bertrand Dunogué, Jérémie Dion, Alexis Régent, Marion Casadevall, Pascal Cohen, Paul Legendre, Benjamin Terrier, Nathalie Costedoat-Chalumeau, Claire Le Jeunne, Luc Mouthon

**Affiliations:** 1 Service de Médecine Interne, Centre de Référence Maladies Autoimmunes Systémiques Rares d’Ile de France, Hôpital Cochin, Assistance Publique-Hôpitaux de Paris (AP-HP), F-75014 Paris, France; ashley.tieu@gmail.com (A.T.); bertrand.dunogue@aphp.fr (B.D.); dion.jeremie@iuct-oncopole.fr (J.D.); alexis.regent@aphp.fr (A.R.); marion.casadevall@aphp.fr (M.C.); pascal.cohen@aphp.fr (P.C.); paul.legendre@aphp.fr (P.L.); benjamin.terrier@aphp.fr (B.T.); nathalie.costedoat@aphp.fr (N.C.-C.); claire.le-jeunne@aphp.fr (C.L.J.); luc.mouthon@aphp.fr (L.M.); 2 APHP-CUP, Hôpital Cochin, Université de Paris, F-75014 Paris, France

**Keywords:** systemic sclerosis, limited, diffuse, anti-centromere antibodies, anti-topoisomerase 1 antibodies

## Abstract

Objective: to describe the prevalences, characteristics, and survivals of patients with anti-topoisomerase 1 antibodies (ATA) and limited cutaneous systemic sclerosis (lSSc) and anti-centromere antibodies (ACA) and diffuse cutaneous systemic sclerosis (dSSc). Methods: patients with ATA lSSc or with ACA dSSc were included in a case-control retrospective study. Results: In our cohort of scleroderma, the prevalence of ACA dSSc and ATA lSSc was 1.1% (12/1040) and 8.9% (93/1040), respectively. ACA dSSc patients had less interstitial lung disease (ILD) (5 (41.7) vs. 74 (79.6); *p* < 0.01), more cardiac involvement, and more muscle involvement (3 (25) vs. 4 (4.3); *p* = 0.03 and 4 (33.3) vs. 4 (7.5); *p* = 0.02,) than ATA dSSc patients. ATA lSSc patients had a higher modified Rodnan skin score than ACA lSSc patients (4 [2–7.5] vs. 2 [0–5]; *p* < 0.01) and less cardiac or muscle involvement than ATA dSSc patients (6 (6.5) vs. 19 (20.4%); *p* < 0.01 and 15 (16.1) vs. 54 (58.1); *p* < 0.0001, respectively). The cumulative 5-year survival rate was 71% in ACA dSSc patients, 95% in ATA lSSc patients, 84% in ACA lSSc patients, and 66% in ATA dSSc patients (*p* < 0.0001). Conclusion: ATA lSSc and ACA dSSc have specific characteristics when compared to ATA dSSc or ACA lSSc. ATA lSSc patients have more ILD than ACA lSSc patients, and ATA dSSc patients have the worst prognosis. Overall, inverted phenotypes show the value of a patient assessment combining antibody and skin subset and should be considered as a separate group.

## 1. Introduction

Systemic sclerosis (SSc) is a systemic autoimmune disease characterized by vascular hyperreactivity, autoimmunity, and fibrosis affecting mainly the skin, the lungs, and the gastrointestinal tract [1]. There are two main clinical phenotypes of SSc: limited SSc (lSSc) and diffuse SSc (dSSc), as defined by the extent of skin involvement [2], which is difficult to assess [3,4,5]. Such a dichotomy is useful in the clinics as limited SSc is associated with less extensive skin involvement, less frequent organ involvement, and a better survival than dSSc [6], but SSc antibodies may also indicate specific involvement [7].

In the American College of Rheumatology/European League Against Rheumatism (ACR/EULAR) set of classification criteria, the three following autoantibodies (Ab) were included: anti-topoisomerase I Ab (ATA), anti-centromere Ab (ACA), and anti-RNA polymerase III Ab. These main Ab are commonly associated with specific features of SSc. ACA are commonly found in lSSc patients and are associated with a better prognosis than dSSc. Still, ACA are also associated with late development of pulmonary hypertension (PH), as well as digital ulcers and gastrointestinal involvement [8]. ATA are commonly associated with dSSc and a poorer prognosis [9,10,11,12]. Indeed, patients with ATA develop severe organ involvement such as severe interstitial lung disease (ILD), cardiac, or renal involvement [8,13]. Lastly, anti-RNA polymerase III Ab (POL3) are found in patients with dSSc and are associated with a high frequency of scleroderma renal crisis, rapidly progressive skin fibrosis, and little pulmonary and gastrointestinal involvement [14]. Despite these common associations between Ab and clinical characteristics, inverted phenotypes of SSc (i.e., ACA dSSc and ATA lSSc) have been described in few studies. In these studies, authors reported that patients with inverted phenotypes had a mild course of SSc [8,15,16,17,18,19]. Still, little is known about the presentation and survival of patients with inverted phenotypes of SSc.

Therefore, the objective of the present study was to characterize for the first time ACA dSSc and ATA lSSc patients followed in a French national referral center for SSc.

## 2. Patients and Methods

### 2.1. Patients 

We performed a monocentric retrospective case-control study, based on the database of the French national referral center for autoimmune and systemic disease, opened since 2000 [20]. Inclusion criteria comprised a diagnosis of SSc according to ACR/EULAR classification criteria [7], ≥18 years of age, with a single Ab (ATA or ACA).

### 2.2. Study Population

Patients were defined as having lSSc or dSSc based on Leroy and Medsger classification [10]. Cases were defined as having ATA lSSc or ACA dSSc if they had lSSc with ATA Ab or dSSc with ACA Ab, respectively. For each case with ATA lSSc, a control with ATA dSSc and a control with ACA lSSc were randomly extracted from the same database of the national referral center for systemic autoimmune diseases of Ile de France and were also used as controls for patients with ACA dSSc.

### 2.3. Objectives of the Study

The primary objective of the study was to determine the prevalence of ACA dSSc and ATA lSSc. Secondary objectives were to describe the clinical characteristics and to study the survival rates of ACA dSSc and ATA lSSc patients.

### 2.4. Data Source

Data included demographic statement (age, gender), disease duration, and clinical profiles (modified Rodnan skin score (mRSS), presence of digital vasculopathy or other visceral impairment such as gastrointestinal tract, interstitial lung disease, pulmonary hypertension, cardiac, and renal). Results of biological samples, pulmonary function test, and echocardiography were also collected for patients and controls. Treatment usage including immunomodulating agents (mycophenolate mofetil, azathioprine, cyclophosphamide, and corticosteroids) were also collected.

Organ system involvement was defined as previously described [21]. Respiratory failure was defined by one of the following three criteria: PaO_2_ < 60 mmHg, or pCO_2_ > 50 mmHg without supplemental oxygen, or resting O_2_ saturation of <88% as determined by pulse oximetry. ILD was defined by pulmonary function tests showing a decrease of >15% in diffusing capacity of the lung of carbon monoxide (DLCO) or >10% in forced vital capacity (FVC) (actual change in % predicted units from baseline). The worsening of mRSS was defined as an increase of mRSS by ≥5 points for skin score or increase by >25% for baseline skin score > 20. Scleroderma renal crisis was defined by high blood pressure and one of the following 5 features: increases of ≥50% above baseline in serum creatinine; proteinuria: ≥2+ by dipstick confirmed by protein/creatinine ratio; hematuria: ≥2+ by dipstick or >10 red blood cells per high power field (without menstruation); thrombocytopenia: <100,000 platelets/mm^3^; hemolysis: by blood smear or increased reticulocyte count. Cardiac involvement was defined by arrhythmia for >3 months, pericarditis, or cardiac heart failure. Myositis was defined by myositis (by elevated CPK and by electromyography (EMG) and/or biopsy). PH was defined as pulmonary artery peak systolic pressure (PAP) of ≥40 mmHg estimated by echocardiography. Joint involvement was defined as inflammatory arthralgias, arthritis, or tendon friction. Calcinosis was identified on hand by X-rays or when it was clinically obvious. Digital tip ulcers were based on physical observation. Gastrointestinal tract involvement was defined by one of the following features: distal esophageal dysmotility, hypomotility of the duodenum or small bowel intestine, malabsorption syndrome, or colon sacculations.

### 2.5. Statistical Analysis

Data for continuous variables are presented as the median and interquartile range (IQR). Data for qualitative variables are presented as the number and percentage. Fisher’s exact test was used to compare qualitative variables, the nonparametric Mann–Whitney U test was used to compare continuous variables, and the *p*-value was corrected by Bonferroni. Kaplan–Meier survival curves were used for the analysis of survival. P values less than 0.05 were considered significant. All analyses were performed using GraphPad Prism 8 or Stata. 

## 3. Results

### 3.1. Prevalence

**Figure 1:**
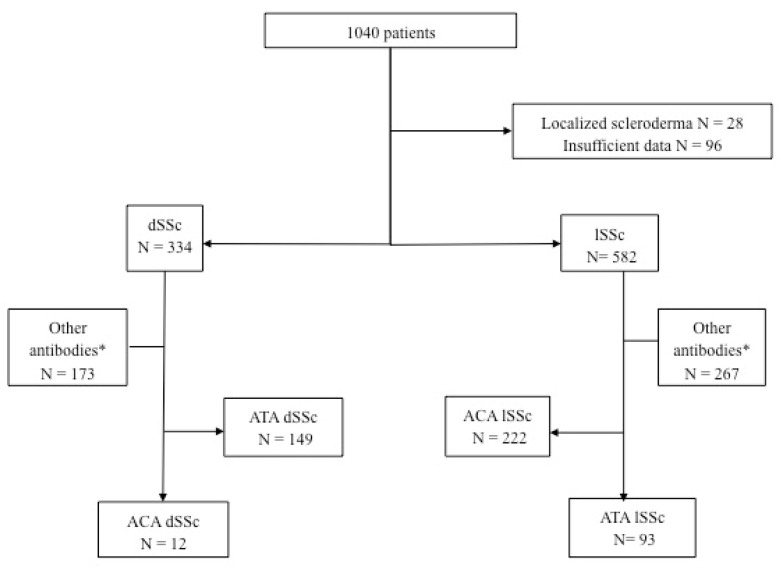
Flow chart of the study. * Other antibodies comprised patients without antibodies and with excluded antibodies (anti-RNA polymerase III, anti-U3 RNP, anti-PM Scl). ACA: anti-centromere antibodies; ATA: anti-topoisomerase 1 antibodies; dSSc: diffuse systemic sclerosis; lSSc: limited systemic sclerosis.

Between 2000 and 2019, 1040 patients with scleroderma were included (Figure 1). Among them, 334 patients had dSSc and 582 patients had lSSc. Among patients with lSSc, 93 had ATA Ab, and among patients with dSSc, 12 had ACA. 

In patients with scleroderma, the prevalence of ACA dSSc was 1.1% (12/1040) and 3.6% (12/334) in patients with dSSc. The prevalence of ATA lSSc was 8.9% (93/1040) in patients with SSc and 16% (93/582) in patients with lSSc (Figure 1).

### 3.2. ACA dSSc Patients

Baseline characteristics of ACA dSSc patients are depicted in Table 1 and Table 2. Demographic characteristics of ACA dSSc subjects were similar to those of ACA lSSc patients and ATA dSSc patients. As expected, organ involvement differed between these three groups.

Compared to ACA lSSc patients, ACA dSSc patients were more severe as they more frequently had skin sclerosis, as evaluated by mRSS (26 [17–30] vs. 2 [7–25]; *p* < 0.01), calcinosis (58% vs. 20%; *p* < 0.05) gastrointestinal tract involvement (92% vs. 58%; *p* < 0.05), heart involvement (3 (25%) vs. 4 (4%); *p* < 0.05), muscle involvement (4 (33%) vs. 4 (8%); *p* < 0.05), inflammatory syndrome (CRP > 5 mg/l) (9 (75%) vs. 14 (5%); *p* < 0.001), and more frequently received immunosuppressants (5 (42%) vs. 5 (5%); *p* < 0.01). 

Compared to ATA dSSc, ACA dSSc patients were quite similar patients but more frequently had calcinosis (58% vs. 16%; *p* < 0.01) and less frequently had ILD (42% vs. 80%; *p* < 0.01). 

After a median follow up of 5 [5–9] years, three (43%) patients with ACA dSSc and eight (12%) patients with ACA lSSc died. The differences in organ involvement, identified at baseline, persisted during the follow-up with a higher mRSS in ACA dSSc than in ACA lSSc patients (23 [22–24] vs. 2 [0–5]; *p* < 0.0001). In ACA dSSc, ILD remained less frequently detected than in ATA dSSc patients (14% vs. 56%; *p* = 0.05).

### 3.3. ATA lSSc Patients

The baseline characteristics of ATA lSSc patients are depicted in Table 3 and Table 4. Compared to ATA dSSc patients, ATA lSSc patients more frequently had an older age at diagnosis of SSc (51 [41–61] vs. 43 [29–54]; *p* < 0.01) and digital ulcers (55 (59%) vs. 34 (37%); *p* < 0.01) but less skin sclerosis as assessed by median (IQR) mRSS (4 [2–8] vs. 18 [10–27]; *p* < 0.0001) and less frequent gastrointestinal tract (59 (60%) vs. 75 (81%); *p* < 0.01), joint (58 (62%) vs. 80 (86%); *p* < 0.001), cardiac (6 (7%) vs. 19 (20%); *p* < 0.01), and muscle involvement (15 (16%) vs. 54 (58%); *p* < 0.0001).

Compared to ACA lSSc patients, ATA lSSc patients had less calcinosis (6 (7%) vs. 22 (23%); *p* < 0.01) and less telangiectasia (27 (29%) vs. 43 (46%); *p* < 0.05). 

Interestingly, ILD was more prevalent in ATA lSSc than in ACA lSSc patients (67 (72%) vs. 31 (33%); *p* < 0.001), whereas it was equally prevalent in ATA dSSc patients (67 (72%) vs. 74 (80%); *p* = 0.30). Although, ATA dSSc more often had a decreased DLCO than ATA lSSc patients (43 (46%) vs. 59 (63%); *p* < 0.01), and the median (IQR) FCV was higher in ATA lSSc (86 [66–103] vs. 71 [60–90]; *p* < 0.01) than in ATA dSSc patients. 

During a median (IQR) follow-up of 5 [3–9] years following inclusion, the median (IQR) mRSS remained lower in ATA lSSc patients than in ATA dSSc patients (4 [2–9] vs. 16 [2–22]; *p* < 0.05), without worsening (*p* = 0.79). Oppositely, the median (IQR) mRSS was similar between ATA lSSc and ACA lSSc patients (4 [2–9] vs. 2 [0–5]; *p* = 0.19). ATA lSSc patients had more ILD than ACA lSSc patients (37 (64%) vs. 7 (10%); *p* < 0.0001) but no less than ATA dSSc patients (37 (64%) vs. 39 (56%); *p* = 0.37). Still, ATA dSSc patients had more severe ILD than ATA lSSc patients, as highlighted by the lower median (IQR) DLCO (42% [34–55] vs. 64% [44–73]; *p* < 0.001), FCV (68% [48–84] vs. 87 [67–99]; *p* < 0.01), and a lower total lung capacity (TLC) (77% [59–87] vs. 90% [71–101]; *p* < 0.001). Other differences during follow-up are depicted in Table 5 and Table 6.

### 3.4. Survival and Transition

**Figure 2:**
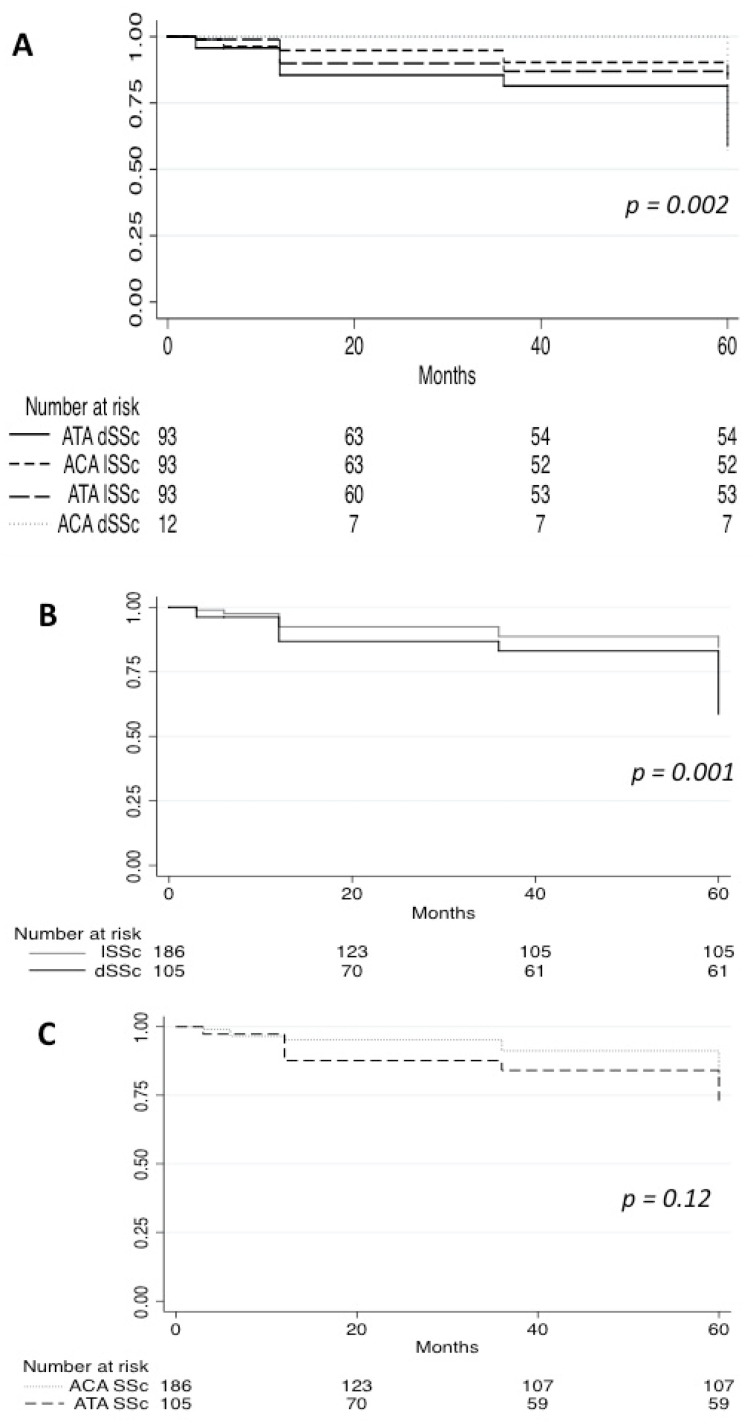
Five-year survival of SSc patients. (**A**): Survival curves of ACA dSSc, ATA dSSc, ACA lSSc, and ATA lSSc. (**B**): Survival curves of dSSc and lSSc patients. (**C**): Survival curves of ACA and ATA SSc patients. ACA: anti-centromere antibodies; ATA: anti-topoisomerase 1 antibodies; dSSc: diffuse systemic sclerosis; lSSc: limited systemic sclerosis.

Survival analysis was undertaken among subjects with at least 1 year of follow-up data (n = 203 patients). The cumulative survival rate during 5 [3–9] years of follow-up was 71% in ACA dSSc and 95% in ATA lSSc compared with 84% (11 patients) in ACA lSSc and 66% (24 patients) in ATA dSSc (Figure 2). Analysis of survival showed a statistically significant (*p* < 0.001) difference between the four groups. Interestingly, survival did not differ significantly between patients with ATA and ACA Ab (*p* = 0.12), whereas patients with dSSc had a worse survival than patients with lSSc (*p* < 0.001).

Of note, none of the lSSc patients of our cohort experienced a transition from limited to diffuse skin involvement.

## 4. Discussion

The objectives of this study were to determine the prevalence and the main characteristics of SSc patients with inverted phenotypes in a French cohort of patients with SSc. According to our results, ACA dSSc and ATA lSSc exist and are rare SSc phenotypes and have their own specificities. Among inverted phenotypes, ACA dSSc is even rarer than ATA lSSc. Interestingly, our study highlights that ATA is specifically associated with ILD and confirms that dSSc has a worse prognosis both in term of organ involvement and mortality.

The prevalence of ACA dSSc and ATA lSSc varies in the literature. In comparison with the two only other published studies (Table 7), we found a lower prevalence of patients with ATA lSSc and a higher prevalence of ACA dSSc [15,16]. At baseline, our patients with inverted phenotypes had a specific clinical phenotype: more ILD in ATA lSSc patients and more cardiac and muscle involvements in ACA dSSc. Regarding follow up, our patients seem to be similar to those reported in the two other studies. Our study also confirms that ACA dSSc and ATA lSSc have a mild course compared to usual phenotypes (ACA lSSc and ATA dSSc) over the 5 years of follow up. As previously reported, lung involvement of patients with ATA lSSc resembles those of ATA dSSc, but ATA lSSc patients have a better survival [15,16]. While the cardiac or muscle involvement or inflammatory syndrome of ACA dSSc patients is similar to those of ATA dSSc, ACA dSSc patients have a better survival than ATA dSSc patients in our study. This finding may reflect the protective role of ACA, suggested by Caetano et al. [22] and also proposed by Srivastava et al., who did not find significative difference between the survival of ACA dSSc and ACA lSSc [16]. 

Our findings confirmed a relationship between Ab and organ involvement. Our data suggest an association between ATA and ILD, whatever the skin subset confirming previous findings [15,16,17,23]. Kranenburg et al. have compared ATA patients with non-ATA patients and showed that ILD was more frequent in ATA dSSc and ATA lSSc than in non-ATA patients [15]. Steen et al. and Walker et al. have suggested that Ab may be a predictor of organ involvement and disease outcome [8,9]. Steen et al. showed that PH typically occurs in patients with ACA and ILD in patients with ATA [8]. This specific lung involvement might be the consequence of a mechanism involving B cells. Indeed, Dumoitier et al. showed a significant proportion of active lymphocyte B in patients with versus those without interstitial lung disease [24], and Fava et al. showed an increased population of ATA-reactive T cells in patients with ILD compared to those without ILD [25]. Other examples were described regarding Ab specificity and clinical presentation. Anti-PM/Scl antibodies are associated with myositis, calcinosis, acro-osteolysis, and interstitial lung disease [26], and the anti-U1RNP antibodies characterize overlapping forms between SSc, systemic lupus erythematosus, and myositis [8].

Furthermore, our study brings data to the relationships between prognosis, antibodies, and skin subsets. Old studies reported a gradient of clinical phenotypes in SSc based on skin involvement. For example, Cottrell et al. found an “intermediate” clinical phenotype (distal to elbow/knees without trunk involvement) with different autoantibody profiles and mild survival [27]. Recent studies have tackled this issue showing that skin extension evaluation could not be sufficient to classify patients. Indeed, Sobanski et al. has determined six different clusters based on clinical features, autoantibody profiles, and survival [28]. In other autoimmune diseases such as ANCA-associated vasculitis, it was showed that patients could be differentiated solely based on their antibody profiling [29]. Although skin extension is a major issue in terms of survival in SSc patients, we believe that our work argues for an evaluation of SSc patients based on both mRSS and autoantibody profile.

Our study has some limitations. As a monocentric study, the number of patients with underrepresented SSc phenotypes such as ACA dSSc is low, and one has to be cautious when looking at characteristics of this subgroup. Since it is a retrospective study, missing data cannot be avoided. Lastly, as a study performed in a tertiary referral center, it included mostly patients with more than 5 years of disease evolution, which limited our ability to study transition patients in depth, as such a phenomenon is reported to occur in the first five years of evolution [15,17].

To conclude, ACA dSSc and ATA lSSc exist and represent 10% of our SSc patient cohort. They are rare phenotypes of SSc, have their own specificities, and are characterized by a mild course of evolution of SSc. Studying these phenotypes confirmed that antibodies assessment in SSc patients is mandatory and that antibodies may predict organ involvement, whereas the extent of skin sclerosis may predict survival. Overall, inverted phenotypes should be considered as a separate group and be assessed mainly combining Ab and skin subset.

## Figures and Tables

**Table 1:** Characteristics of patients with anti-centromere antibodies (ACA).

Characteristics	Diffuse (N = 12)		Limited (N = 93)		*p*
Females	8	(66.7)	82	(88.2)	0.07
Age at diagnostic, years, median (IQR)	40	[31–57]	54	[32–56]	0.83
Age at baseline, years, median (IQR)	50	[40–64]	59	[38–61]	0.88
Modified Rodnan skin score, median (IQR)	26	[17–30]	2	[7–25]	<0.01
Mouth opening, mm, median, (IQR)	30	[19–39]	40	[35–42]	0.22
Calcinosis	7	(58.3)	22	(20)	0.02
Telangiectasia	7	(58.3)	43	(46.2)	0.54
Digital ulcers	7	(58.3)	33	(35.5)	0.20
Gastrointestinal tract involvement	11	(91.7)	54	(58.1)	0.02
Joint involvement	8	(66.7)	51	(54.8)	0.54
Tendon friction rubs	0	(0)	0	(0)	-
Pulmonary arterial hypertension	3	(25)	13	(14)	0.39
sPAP, mmHg, median (IQR)	30	[26–37]	30	[26–37]	0.25
Interstitial lung disease	5	(41.7)	31	(33.3)	0.74
DLCO, %, median (IQR)	73	[62–86]	68	[55–76]	0.44
DLCO < 70%	5	(41.7)	35	(37.6)	0.76
FVC, %, median (IQR)	93	[77–97]	102	[81–116]	<0.01
TLC, %, median (IQR)	92	[82–102]	105	[91–117]	0.02
Scleroderma renal crisis	2	(16.7)	2	(2.2)	-
Heart involvement	3	(25)	4	(4.3)	0.03
Muscle involvement	4	(33.3)	7	(7.5)	0.02
Inflammatory syndrome (CRP > 5 mg/L)	9	(75)	14	(15.1)	<0.0001
CRP, median, (IQR)	9	[7.5–17.5]	2	[1–4.5]	-
Immunosuppressant	5	(41.7)	5	(5.4)	<0.01
Mycophenolate mofetil	0	(0)	1	(1.1)	-
Azathioprine	0	(0)	0	(0)	-
Cyclophosphamide	3	(25)	0	(0)	-
Corticosteroids	4	(33.3)	7	(7.5)	0.02
Corticosteroids dose (mg) median (IQR)	10	[10–10]	0	[0]	-

Results are indicated as number (percentage) unless indicated differently. ACA: anti-centromere antibody; CRP: C reactive protein; DLCO: diffusing capacity of the lung of carbon monoxide; FCV: forced vital capacity; IQR: interquartile range; N: number; sPAP: systolic pulmonary artery pressure; PH: pulmonary hypertension; ATA: anti-topoisomerase 1 antibody; TLC: total lung capacity; %: percentage.

**Table 2:** Demographic characteristics of patients with diffuse systemic sclerosis (dSSc).

Characteristics	ACA (N = 12)		ATA (N = 93)		*p*
Females	8	(66.7)	73	(78.5)	0.46
Age at diagnostic, years, median (IQR)	40	[31–57]	43.0	[29–54]	0.44
Age at baseline, years, median (IQR)	50	[40–64]	49.0	[36–60]	0.54
Modified Rodnan skin score, median (IQR)	26	[17–30]	18	[10–27]	0.13
Mouth opening, mm, median, (IQR)	30	[19–39]	30	[25–35]	0.94
Calcinosis	7	(58.3)	15	(16.1)	<0.01
Telangiectasia	7	(58.3)	37	(39.8)	0.23
Digital ulcers	7	(58.3)	55	(59.1)	1
Gastrointestinal tract involvement	11	(91.7)	75	(80.6)	0.69
Joint involvement	8	(66.7)	80	(86)	0.10
Tendon friction rubs	0	(0)	5	(5.4)	1
Pulmonary arterial hypertension	3	(25)	13	(14)	0.39
sPAP, mmHg, median (IQR)	30	[26–37]	32	[28–36]	0.63
Interstitial lung disease	5	(41.7)	74	(80)	<0.01
DLCO, %, median (IQR)	73	[62–86]	50	[28–36]	0.04
DLCO < 70%	5	(41.7)	59	(63.4)	0.21
FVC, %, median (IQR)	93	[77–97]	71	[60–90]	0.42
TLC, %, median (IQR)	92	[82–102]	79	[63–89]	0.24
Scleroderma renal crisis	2	(16.7)	5	(5.4)	0.81
Heart involvement	3	(25)	19	(20.4)	0.71
Muscle involvement	4	(33.3)	54	(58.1)	0.13
Inflammatory syndrome (CRP > 5 mg/L)	9	(75)	47	(50.5)	0.15
CRP, median, (IQR)	9	[7.5–17.5]	9	[4–17]	0.58
Immunosuppressant	5	(41.7)	18	(19.4)	0.13
Mycophenolate mofetil	0	(0)	1	(1.1)	-
Azathioprine	0	(0)	0	(0)	-
Cyclophosphamide	3	(25)	14	(15.1)	-
Corticosteroids	4	(33.3)	12	(12.9)	-
Corticosteroids dose (mg) median (IQR)	10	[10–10]	11	[8–15]	-

Results are indicated as number (percentage) unless indicated differently. ACA: anti-centromere antibody; ATA: anti-topoisomerase 1 antibody; CRP: C reactive protein; DLCO: diffusing capacity of the lung of carbon monoxide; FCV: forced vital capacity; IQR: interquartile range; N: number; sPAP: systolic pulmonary artery pressure; PH: pulmonary hypertension; TLC: total lung capacity; %: percentage.

**Table 3:** Characteristics of patients with anti-topoisomerase 1 antibodies (ATA).

Characteristics	Diffuse (N = 93)		Limited (N = 93)		*p*
Females	73	(78.5)	77	(82.8)	0.58
Age at diagnostic, years, median (IQR)	43.0	[29–54]	51	[41–61]	<0.01
Age at baseline, years, median (IQR)	49.0	[36–60]	56	[48–67]	<0.001
Modified Rodnan skin score, median (IQR)	18	[10–27]	4	[2–7.5]	<0.0001
Mouth opening, mm, median, (IQR)	30	[25–35]	38	[35–40]	<0.0001
Calcinosis	15	(16.1)	6	(6.5)	0.06
Telangiectasia	37	(39.8)	27	(29)	0.16
Digital ulcers	55	(59.1)	34	(36.6)	<0.01
Gastrointestinal tract involvement	75	(80.6)	56	(60.2)	<0.01
Joint involvement	80	(86)	58	(62.4)	<0.001
Tendon friction rubs	5	(5.4)	4	(4.3)	1
Pulmonary arterial hypertension	13	(14)	22	(23.7)	0.13
sPAP, mmHg, median (IQR)	32	[28–36]	33	[29–45]	0.16
Interstitial lung disease	74	(80)	67	(72)	0.30
DLCO, %, median (IQR)	50	[28–36]	60	[41–77]	0.26
DLCO < 70%	59	(63.4)	43	(46.2)	0.03
FVC, %, median (IQR)	71	[60–90]	86	[66–103]	<0.01
TLC, %, median (IQR)	79	[63–89]	85	[67–103]	0.05
Scleroderma renal crisis	5	(5.4)	8	(8.6)	0.57
Heart involvement	19	(20.4)	6	(6.5)	<0.01
Muscle involvement	54	(58.1)	15	(16.1)	<0.0001
Inflammatory syndrome (CRP > 5 mg/L)	47	(50.5)	28	(30.1)	<0.01
CRP, median, (IQR)	9	[4–17]	4	[2–10]	0.2
Immunosuppressant	18	(19.4)	13	(14)	0.43
Mycophenolate mofetil	1	(1.1)	1	(1.1)	-
Azathioprine	0	(0)	2	(2.2)	-
Cyclophosphamide	14	(15.1)	6	(6.5)	-
Corticosteroids	12	(12.9)	5	(5.4)	-
Corticosteroids dose (mg) median (IQR)	11	[8–15]	10	[5–15]	-

Results are indicated as number (percentage) unless indicated differently. ATA: anti-topoisomerase 1 antibody; CRP: C reactive protein; DLCO: diffusing capacity of the lung of carbon monoxide; FCV: forced vital capacity; IQR: interquartile range; N: number; sPAP: systolic pulmonary artery pressure; PH: pulmonary hypertension; TLC: total lung capacity; %: percentage.

**Table 4:** Demographic characteristics of patients with limited systemic sclerosis (lSSc).

Characteristics	ACA (N = 93)		ATA (N = 93)		*p*
Females	82	(88.2)	77	(82.8)	0.41
Age at diagnostic, years, median (IQR)	54	[32–56]	51	[41–61]	0.34
Age at baseline, years, median (IQR)	59	[38–61]	56	[48–67]	0.42
Modified Rodnan skin score, median (IQR)	2	[7–25]	4	[2–7.5]	<0.01
Mouth opening, mm, median, (IQR)	40	[35–42]	38	[35–40]	0.51
Calcinosis	22	(23)	6	(6.5)	<0.01
Telangiectasia	43	(46.2)	27	(29)	0.02
Digital ulcers	33	(35.5)	34	(36.6)	1
Gastrointestinal tract involvement	54	(58.1)	56	(60.2)	0.88
Joint involvement	51	(54.8)	58	(62.4)	0.37
Tendon friction rubs	0	(0)	4	(4.3)	0.12
Pulmonary arterial hypertension	13	(14)	22	(23.7)	0.13
sPAP, mmHg, median (IQR)	30	[26–37]	33	[29–45]	<0.01
Interstitial lung disease	31	(33.3)	67	(72)	<0.0001
DLCO, %, median (IQR)	68	[55–76]	60	[41–77]	0.44
DLCO < 70%	35	(37.6)	43	(46.2)	0.30
FVC, %, median (IQR)	102	[81–116]	86	[66–103]	0.03
TLC, %, median (IQR)	105	[91–117]	85	[67–103]	<0.01
Scleroderma renal crisis	2	(2.2)	8	(8.6)	0.10
Heart involvement	4	(4.3)	6	(6.5)	0.75
Muscle involvement	7	(7.5)	15	(16.1)	0.11
Inflammatory syndrome (CRP > 5 mg/L)	14	(15.1)	28	(30.1)	0.02
CRP, median, (IQR)	2	[1–4.5]	4	[2–10]	<0.01
Immunosuppressant	5	(5.4)	13	(14)	0.08
Mycophenolate mofetil	1	(1.1)	1	(1.1)	-
Azathioprine	0	(0)	2	(2.2)	-
Cyclophosphamide	0	(0)	6	(6.5)	-
Corticosteroids	7	(7.5)	5	(5.4)	-
Corticosteroids dose (mg) median (IQR)	0	[0]	10	[5–15]	-

Results are indicated as number (percentage) unless indicated differently. ACA: anti-centromere antibody; ATA: anti-topoisomerase 1 antibody; CRP: C reactive protein; DLCO: diffusing capacity of the lung of carbon monoxide; FCV: forced vital capacity; IQR: interquartile range; N: number; sPAP: systolic pulmonary artery pressure; PH: pulmonary hypertension; TLC: total lung capacity; %: percentage.

**Table 5:** Characteristics of patients with limited systemic sclerosis at last follow-up.

Characteristics	ACA (N = 68)		ATA (N = 58)		*p*
Follow-up, years, median (IQR)	5	[3–9]	5	[3–9]	0.64
Death	8	(11.8)	3	(5.2)	0.23
Worsening of modified Rodnan skin score	1	(1.5)	6	(10.3)	0.05
Modified Rodnan skin score, median (IQR)	2	[0–5]	4	[2–9]	0.19
Mouth opening, mm, median (IQR)	40	[34–42]	37	[33–41]	0.30
Calcinosis	9	(13.2)	3	(5.2)	0.14
Telangiectasia	22	(32.4)	11	(19)	0.11
Digital ulcers	11	(16.2)	13	(22.4)	0.50
Gastrointestinal tract involvement	35	(51.5)	15	(25.9)	<0.01
Joint involvement	10	(14.7)	9	(15.5)	1.00
Tendon friction rubs	0	(0)	0	(0)	1.00
New onset pulmonary arterial hypertension	4	(5.9)	0	(0)	0.12
Pulmonary arterial hypertension	11	(16.2)	4	(6.9)	0.17
Systolic pulmonary artery pressure, mmHg, median (IQR)	30	[28–38]	29	[25–33]	0.14
New onset respiratory failure	0	(0)	0	(0)	1.00
Worsening of DLCO	10	(14.7)	18	(31)	0.03
Worsening of FVC	7	(10.3)	9	(15,5)	0.43
Interstitial lung disease	7	(10.3)	37	(63,8)	<0.001
DLCO, %, median (IQR)	70	[62–76]	64	[44–73]	0.03
DLCO < 70%	19	(27.9)	22	(37.9)	0.70
FVC, %, median (IQR)	107	[91–118]	87	[67–99]	<0.001
TLC, %, median (IQR)	108	[101–118]	90	[71–101]	<0.001
New onset scleroderma renal crisis	3	(4.4)	0	(0)	0.25
New onset heart involvement	3	(4.4)	4	(6.9)	0.70
New onset muscle involvement	7	(10.3)	6	(10.3)	1.00
Inflammatory syndrome (CRP > 5 mg/L)	12	(17.6)	10	(17.2)	1.00
CRP, median (IQR)	1.6	[1–7.9]	1.5	[0–7.1]	0.60
Immunosuppressant	1	(1.5)	15	(25.9)	<0.0001

Results are indicated as number (percentage) unless indicated differently. ACA: anti-centromere antibody; ATA: anti-topoisomerase 1 antibody; CRP: C reactive protein; DLCO: diffusing capacity of the lung of carbon monoxide; FCV: forced vital capacity; IQR: interquartile range; N: number; sPAP: systolic pulmonary artery pressure; PH: pulmonary hypertension; TLC: total lung capacity; %: percentage.

**Table 6:** Characteristics of patients with anti-topoisomerase 1 antibodies at last follow-up.

Characteristics	Diffuse (N = 70)		Limited (N = 58)		*p*
Follow-up, years, median (IQR)	7.5	[4–12]	5	[3–9]	0.08
Death	21	(30)	3	(5.2)	<0.001
Worsening of modified Rodnan skin score	9	(12.9)	6	(10.3)	0.79
Modified Rodnan skin score, median (IQR)	16	[2–22]	4	[2–9]	0.03
Mouth opening, mm, median (IQR)	29	[1–35]	37	[33–40]	<0.0001
Calcinosis	6	(8.6)	3	(5.2)	0.51
Telangiectasia	15	(21.4)	11	(19)	0.83
Digital ulcers	29	(41.4)	13	(22.4)	0.02
Gastrointestinal tract involvement	26	(37.1)	15	(25.9)	0.19
Joint involvement	14	(20)	9	(15.5)	0.64
Tendon friction rubs	0	(0)	0	(0)	-
New onset pulmonary arterial hypertension	8	(11.4)	0	(0)	0.13
Pulmonary arterial hypertension	14	(20)	4	(6.9)	0.04
Systolic pulmonary artery pressure, mmHg, median (IQR)	36	[30–44]	29	[25–33]	<0.01
New onset respiratory failure	0	(0)	0	(0)	-
Worsening of DLCO	25	(35.7)	18	(31)	0.71
Worsening of FVC	12	(17.1)	9	(15.5)	1.00
Interstitial lung disease	39	(55.7)	37	(63.8)	0.37
DLCO, %, median (IQR)	42	[34–55]	64	[44–73]	<0.01
DLCO < 70%	30	(42.9)	22	(37.9)	0.59
FVC, %, median (IQR)	68	[48–84]	87	[67–99]	<0.01
TLC, %, median (IQR)	77	[59–87]	90	[71–101]	<0.01
New onset scleroderma renal crisis	4	(5.7)	0	(0)	0.13
New onset heart involvement	7	(10)	4	(6.9)	0.75
New onset muscle involvement	8	(11.4)	6	(10.3)	1.00
Inflammatory syndrome (CRP > 5 mg/L)	19	(27.1)	10	(17.2)	0.21
CRP, median (IQR)	2	(0–15)	2	[0–7]	0.24
Immunosuppressant	30	(42.9)	15	(25.9)	0.06

Results are indicated as number (percentage) unless indicated differently. CRP: C reactive protein; DLCO: diffusing capacity of the lung of carbon monoxide; FCV: forced vital capacity; IQR: interquartile range; N: number; sPAP: systolic pulmonary artery pressure; PH: pulmonary hypertension; TLC: total lung capacity; %: percentage.

**Table 7:** Reported series of inverted phenotypes in systemic sclerosis patients.

		ACA dSSc		ATA lSSc		
	Srisvastava et al.	Kranenburg et al.	Tieu et al.	Srisvastava et al.	Kranenburg et al.	Tieu et al.
	2015	2016	2022	2015	2016	2022
General characteristics						
Patients, N	91	87	12	52	58	93
Prevalence (%)	16.5	18.9	4.1	9.4	12.6	32
Baseline characteristics						
Interstitial lung disease (ILD)	19 (22.1)	15 (17.2)	5 (41.7)	25 (49.0)	17 (29.3)	67 (72)
Pulmonary arterial hypertension (PAH)	13 (16.3)	6 (6.9)	3 (25)	2 (4.8)	1 (1.7)	22 (23.7)
Cardiac involvement		4 (4.6)	3 (25)	-	4 (6.9)	6 (6.5)
Scleroderma renal crisis	0 (0.0)	5 (5.7)	2 (16.7)	2 (3.8)	0 (0.0)	8 (8.6)
Myositis	4 (4.4)	-	4 (33.3)	4 (7.7)	-	15 (16.1)
Joint involvement	29 (33.0)	-	8 (66.7)	14 (27.5)	-	58 (62.4)
Follow up						
Patients with ≥1 year of follow up, N	-	-	7	-	-	58
ILD	-	28 (38.9)	1 (14.3)	-	20 (48.8)	37 (63.8)
PAH	-	2 (2.5)	2 (28.6)	-	4 (7.0)	5 (8.6)
Cardiac involvement	-	11 (13.3)	1 (14.3)	-	6 (11.1)	3 (5.2)
Scleroderma renal crisis	-	7 (8.5)	1 (14.3)	-	0 (0.0)	0 (0)
Myositis	-	-	0 (0.0)	-	-	6 (10.3)
Joint involvement	-	-	1 (14.3)	-	-	10 (17.2)
Survival						
Death	-	18 (20.7)	3 (42.9)	-	6 (10.3)	3 (5.2)

Results are indicated as number (percentage) unless indicated differently. ACA: anti-centromere antibody; ATA: anti-topoisomerase 1 antibody; ILD: interstitial lung disease; N: number; PAH: pulmonary arterial hypertension; SSc: systemic sclerosis; %: percentage.

## Data Availability

Data are available on demand to Dr. Benjamin Chaigne.

## Abbreviations

Ab	Antibody
ACA	Anti-centromere antibody
ACR/EULAR	American College of Rheumatology/European League Against Rheumatism
ATA	Anti-topoisomerase 1 antibody
CRP	C reactive protein
DLCO	Diffusing capacity of the lung of carbon monoxide
dSSc	Diffuse systemic sclerosis
FVC	Forced vital capacity
ILD	Interstitial lung disease
IQR	Interquartile range
lSSc	Limited systemic sclerosis
mRSS	Modified Rodnan skin score
PAP	Pulmonary artery pressure
PH	Pulmonary hypertension
POL3	Anti-RNA polymerase III antibody
SSc	Systemic sclerosis
TLC	Total lung capacity
